# Machine Learning Models for Predicting and Classifying the Tensile Strength of Polymeric Films Fabricated via Different Production Processes

**DOI:** 10.3390/ma12091475

**Published:** 2019-05-07

**Authors:** Safwan Altarazi, Rula Allaf, Firas Alhindawi

**Affiliations:** Industrial Engineering Department, German Jordanian University, Amman 11180, Jordan; rula.alalf@gju.edu.jo (R.A.); firas.alhindawi@gju.edu.jo (F.A.)

**Keywords:** machine learning algorithms, polymeric films, extrusion-blow molding, cryomilling-compression molding

## Abstract

In this study, machine learning algorithms (MLA) were employed to predict and classify the tensile strength of polymeric films of different compositions as a function of processing conditions. Two film production techniques were investigated, namely compression molding and extrusion-blow molding. Multi-factor experiments were designed with corresponding parameters. A tensile test was conducted on samples and the tensile strength was recorded. Predictive and classification models from nine MLA were developed. Performance analysis demonstrated the superior predictive ability of the support vector machine (SVM) algorithm, in which a coefficient of determination and mean absolute percentage error of 96% and 4%, respectively were obtained for the extrusion-blow molded films. The classification performance of the MLA was also evaluated, with several algorithms exhibiting excellent performance.

## 1. Introduction

Polymeric materials in the form of films have found numerous technological applications in various industrial and biomedical sectors. Films are continuous layers of polymers up to 0.3 mm thick (thicker layers are called sheets). Polymeric films are made from natural and synthetic polymers. Almost all plastics can be formed into films. In many instances, films have complex compositions with different blends of polymers and fillers such as electrical conductive substances, pigments, and nanoparticles, in addition to different structures and textures. Various technologies have been utilized to fabricate films with different thicknesses and properties. The oldest technology in plastic film manufacturing is solvent casting; after 1950, film extrusion techniques of thermoplastic polymers became dominant [1]. Rolling (calendering), drawing, or blowing operations may follow extrusion to reduce film thickness and improve its strength [2,3]. Recently, several techniques emerged to produce thin and ultrathin films, such as dip-coating, spray-coating, spin-coating, self-assembly, layer-by-layer assembly, and several deposition techniques. These processes involve much more than physical shaping of the polymer; they also influence phase morphology, molecular alignment, crystallinity, etc., and ultimately the performance of the product [4]. Thin polymeric films also exhibit unusual physical properties due to the geometric confinement effects and/or interfacial interactions.

Melt extrusion processes are typically the most convenient, continuous, versatile, economical, and environmentally friendly for film and sheet fabrication [4]. Extruders with rotating screws, which transport the material through a heated barrel past a forming die, are the heart of such processes. Frequently, mixing and compounding are also involved in the process [4]. For film blow molding, a continuous tubing is extruded through an annular die of an extruder. Subsequently, air under controlled positive pressure is blown inside the tube inflating it around a trapped air bubble like a balloon. Besides, the tube is drawn in the axial direction as it emerges from the die. Consequently, the wall thickness is continuously reduced to produce a thin cylindrical film, which can be sealed at the end to make bags, or cut and laid flat to make films [2]. Compression molding, on the other hand, is simple, economical, environmentally friendly, and does not involve material flow and shear forces. Furthermore, it does not involve mixing or compounding, thus a preprocessing operation is required to prepare blend and composite powders for molding. These processes are multi-variable, multi-stage, with non-linear viscous and pressure effects. The parameter settings in the process are often chosen based on the references or handbooks. Subsequently, trial and error runs are required to adjust the settings to obtain the desired output. Lower production cost, shorter development time, reduction in the defects, and improved productivity could be achieved, if statistical and optimization techniques are utilized [5]. Statistical methods such as regression have been widely conducted in numerous works [5]. We aim to investigate MLA for modeling the tensile properties of produced films.

Machine learning (ML) is a branch of artificial intelligence (AI) related to the creation of models (knowledge) that can effectively learn from existing data [6,7,8,9,10]. Over the past decades, ML has developed into a wide and diverse field of research, resulting in a variety of different algorithms, theories, tools, application areas, etc. [11]. However, learning/algorithms have been roughly described into three classes: (i) supervised learning, where learning is based on the comparison of computed output with desired output; the algorithm generates a model that maps inputs to desired outputs. (ii) Unsupervised learning, where learning is merely based on the input pattern; the algorithm is designed to extract structure from data. (iii) Reinforcement learning, where the algorithm learns policies/rules on how to act to generate best results based on trial and error [11,12,13]. Today, the field of ML is so vast and proved useful in many segments of industry and basic sciences. Its algorithms have shown great promise as efficient tools for modeling and classification of complex production processes [5,14,15,16] and materials science problems [17,18,19,20,21,22,23,24]. Compared with conventional statistical modeling techniques, such as linear regression and response surface methodology, AI-based methods have shown superiority as modeling techniques for data sets showing non-linear relationships [25,26]. These techniques have demonstrated surprising capability in recognizing patterns of enormous complexity and capturing complex interactions among input and output variables in a system. They have also shown enormous performance in quantitative structure-property-relationship investigations [23]. 

Prediction and classification of film properties is not a trivial task and deviations from bulk behaviors are common. Film composition, components sizes and percentages, preprocessing, and many other material-based parameters have shown direct effects on film properties [25,26]. Process parameters have also revealed significant effects [27,28]. Polymer processing is in general difficult to predict and model, especially because of the interdependencies between processing conditions, polymeric behaviors, and geometries. Process modelling typically involves significant amount of experimentation along with analytical modeling and/or numerical simulation; such procedures are also cyclical with several trial-and-error runs, costly and time-consuming tests, and material losses [29,30]. With the rapid advancement in soft computing, machine learning algorithms (MLA) have been advocated to solve complex modeling and optimization problems in various engineering fields [16,31]. 

In the present research study, we aim to assess and compare the prediction and classification capability of nine MLA in the polymeric film production field, namely, k-nearest neighbors (kNN); decision tree (DT); artificial neural network (ANN); support vector machine (SVM); AdaBoost (AB); random forest (RF); stochastic gradient descent (SGD); and regression analysis (linear regression (LR) for prediction and logistic regression (LoR) for classification). Generally, these algorithms have several advantages that make them suitable in predicting or classifying quality indices of products produced in a multi-parameter production process, such as the polymer production processes under study. 

## 2. Experimental Dataset Development

This study aimed at developing and comparing MLA predicting and classifying models for the tensile strength of films produced by two different melt processing technologies, namely extrusion blow molding and compression molding, as function of processing parameters and material composition. This section provides the details of laboratory experiments and the data development method.

### 2.1. Extrusion-Blow Molding

Virgin high-density polyethylene (HDPE) was utilized in the extrusion blow molding study. The polymer is a commercial product from SABIC (Riyadh, Saudi Arabia) with a density of 952 kg/m^3^ and a melt flow index of 0.7 g/10 min (at 190 °C with 2.16 kg). Recycled HDPE was also used; it was obtained from a Jordanian recycler. A copolymer was further added to enhance processability and performance of the blow molded films. Finally, CaCO_3_ filler in powder form having two different mean particle sizes (6 and 12 µm), purchased from the Jordanian Calcium Carbonate Co. (Amman, Jordan), was employed as a matrix filler. Before blow molding, the materials were mixed to different formulations with an SHR-10A mixer (Zhangjiagang Jinguan Industry Machinery Co., Ltd., Suzhou, China). The HDPE films were prepared by extrusion blow molding on a Mini Film Blowing Machine (SJ-D50/55, Zhejiang, China) with a single screw extruder with a screw diameter of 25 mm, screw length to diameter ratio 24:1, screw compression ratio of 3:1, and four individually controlled temperature zones. The screw (Figure S1 in the supplementary materials) was tapred in the feed and compression zones but had constant channel depath, of 2.5 mm, in the measuring zone which represents 10% of the screw length. The extruder was equipped with a conventional film-blowing die with a diameter of 60 mm and a film-blowing tower with a calendering nip and takeoff rolls. The blow molding formulation and processing parameters (filler size, temperatures of the extruder’s four heaters, the extruder’s mixing speed, and the bubble drawing up speed) were manipulated according to a Minitab-prepared mixture design with extreme vertices and processing variables set as shown in Table 1, which resulted in a total of 86 experimental runs [32]. Table S1, in the supplementary materials, provide the full experimental data set for the extrusion-blow molding. 

### 2.2. Cryomilling/Compression Molding

Biodegradable polymers, namely poly(ε-caprolactone) (PCL) and poly(ethylene oxide) (PEO) were utilized in the compression molding study. PCL powder (molecular weight, Mn ~ 47.5 kDa), was supplied by Perstorp UK Limited, UK. PEO fine powder (molecular weight, Mv ~ 100,000) was purchased from Sigma-Aldrich, St. Louis, MO, USA. Wood sawdust from the German-Jordanian University workshop was also exploited as a filler in the process.

Prior to molding, PCL/PEO/wood powders of different compositions (Table 2) were cryogenically blended in a Retsch Cryomill (Retsch GmbH, Haan, Germany) for different times (27, 54, 81 min) at a frequency of 30 Hz. Subsequently, powder samples were loaded on a flat steel mold between Teflon sheets and compression molded in a Carver bench-top laboratory press (Carver, Inc., Wabash, IN, USA). The compression molding cycle consisted of the following steps. The press platens were first heated up to the required temperature (100, 125, 150 °C). Once the required temperature was obtained, the mold was placed between the platens and a 50 kN force was applied and maintained for different molding times (~0.5 and/or 5 min). Next, heat was turned off and the mold was cooled down to room temperature utilizing one of three cooling techniques: (a) 30 min machine cooling followed by water circulation, (b) water circulation, (c) liquid nitrogen (LN2) cooling, where the molded film between Teflon sheets was dropped in liquid nitrogen. The number of experminetal runs were 71 with three to four specimens fabricated at each experimental run and data averaged for analysis purposes to enhance the reproducibility of the results. Table S2, in the supplementary materials, provide the full experimental data set for the cryomilling/compression molding.

### 2.3. Measurement of Tensile Strength

After film fabrication, samples were cut into small rectangular pieces (6 mm width) for tensile testing (35 mm gauge length). Thicknesses were measured using a digital micrometer. Tensile strength was calculated at peak stress from stress-strain curves obtained using a Testometric universal tensile testing machine (Testometric Co. Ltd., Rochdale, UK) at a cross-head speed of 1 mm/min and a temperature of 23 °C. Furthermore, tensile modulus was determined by linear regression analysis as the slope of the first linear region of the stress-strain curve, and ductility as percentage elongation at break, expressed as a percent of the gage length.

## 3. MLA Models Development

### 3.1. MLA: Background

In the present research study, we aim to assess and compare the prediction and classification capability of nine learning algorithms in the polymeric film production field, namely, kNN, DT, ANN, SVM, AB, RF, SGD, and regression analysis (LR for prediction and LoR for classification). 

The kNN algorithm is a simple supervised MLA where prediction or classification of a test data is conducted based on its k most correlated neighbors from the training set. The kNN is non-parametric, does not assume linear separability of the data, is very stable and robust to small changes in the data, and can learn from a small set of objects while maintaining a competitive performance [10]. A DT is a decision support tool commonly used in data mining, with a tree-like structure made of nodes and branches. There are many DT algorithms available with classification and regression tree (CART) being one of the most extensively applied [18,33]. CART is adopted in the current research as it can provide insight into the relationships and interactions between the input parameters, thus, can be used in understanding materials behavior [18]. ANN are the most commonly used nonparametric and nonlinear MLA inspired by the behavior of neurons in a brain. It makes use of a number of simple highly interconnected processing elements (nodes or neurons) to process information. Typically, a node performs a linear regression followed by a nonlinear function (activation functions). Nodes are placed in layers and connected with links (weights), such that information flows from nodes on the input layer, through nodes on hidden layer(s), to output nodes [11,12,13,34,35]. In this research, we adopted the multi-layer perceptron class of feedforward ANN, which is a supervised learning algorithm capable of learning nonlinear functions. SVM is also one of the most robust and accurate MLA which can be used for both classification and regression. It is based on statistical learning theory and structural risk minimization (SRM) inductive principal [10]. Basically, the input dataset is mapped into a high dimension feature space and a linear model (hyperplane) is constructed in that space. SVM uses kernel functions, such as radial basis function (RBF), sigmoid, polynomial and linear kernel functions, to determine a hyperplane/line that best separates the dataset into classes [36]. The optimal hyperplane is that which maximizes the margin, i.e., the distance between the hyperplane and the closest data points (support vectors). Ensemble learning is a class of MLA that combine several learners to solve a classification or prediction problem [11,13]. AB is a well-known boosting ensemble, which converts a group of weak learners into a group of strong ones. The RF algorithm is another ensemble-based learning method that belongs to the family of averaging, also called bagging, ensemble methods [31]. Gradient descent are popular optimization algorithms used in ML while training a model. They can be combined with other MLA to minimize a cost function and reach a local minimum by adjusting its parameters. SGD is a simple and efficient algorithm that has been gaining attention recently with the rise up of large-scale learning [37,38]. Regression is also a form of learning based on the relationship between variables obtained from a continuous dataset [39]. Notable statistical regression techniques used in ML are linear and logistic regression. LR evaluates the linear relationship between a dependent variable and one or a group of independent predictors. LoR is similar to LR but is used for classification purposes that uses the sigmoid/logistic function to transform a real-number predicted value to a binary one (0 or 1). 

### 3.2. Model Development

To model the tensile strength of fabricated films, nine supervised MLA were implemented using the open source machine learning and data visualization software Orange3 (Bioinformatics Lab, University of Ljubljana, Ljubljana, Slovenia) [40]. The two datasets were analyzed separately. The first dataset was the extrusion-blow molding set and consisted of 258 samples (these resulted from three replicates at each of the 86 mixture design combinations). The second was the cryomilling/compression molding dataset and consisted of 216 samples (records). For the first dataset, the inputs (predictor variables) consisted of eight variables, which were the weight percentages of virgin HDPE; recycled HPDE; CaCO_3_, and copolymer, the CaCO_3_ mean particle size, the temperatures of the extruder’s heaters (considering the average of four), the extruder’s mixing speed, and the bubble drawing up speed. The inputs for the second dataset consisted of seven variables, which were weight percentages of PCL; PEO; and wood, molding time, molding temperature, milling time, and the cooling technique used. As mentioned before, the quality index (output) considered is the tensile strength (MPa). 

Each dataset was then divided randomly into two sets: training data and testing data, representing ~80% and 20% of the total data, respectively. A 20-fold (k = 20) cross validation procedure was used on the training set to optimize the parameters of the model until attaining a high coefficient of determination (R^2^). Table 3 shows the MLA parameters which resulted in a satisfactory performance measure during cross validation. After building the model using the training data, the testing dataset (20% of the data) was utilized to evaluate the trained model’s performance according to the R^2^ (Equation (1)) and mean absolute percentage error (MAPE) (Equation (2)) criteria, for the regression implementation of the algorithms.

(1) $$R^{2}=1-\frac{SS_{residuals}}{SS_{total}}$$

(2) $$\mathrm{MAPE}=\frac{\sum_{t=1}^{n}\left|\frac{A_{t}-F_{t}}{A_{t}}\right|}{n}\times 100\%,$$

where *SS_residuals_* is the variability unrepresented by the model, *SS_total_* is the total variability in the dataset, *n* is the number of tested data instances, *F_t_* is the predicted value estimated by the model for instance data *i*, and *A_t_* is the target (actual) value of the instance data *i*.

The classification ability of the MLA was examined for the extrusion-blow molding process. The data was classified into two categories; “conforming” or “nonconforming” in accordance with the minimum specification limit for the tensile strength as 3500 psi (24.13 MPa) set by the ASTM D-882 standard [41]. In view of this standard, the percentage of nonconforming films (18%) in the studied dataset was significantly lower than the conforming percentage; which resulted in an imbalanced dataset. MLA are prone to produce faulty classifiers when trained on imbalanced datasets, since they tend to treat the minority class as noise in the dataset. Accuracy, defined here as the proportion of correctly classified instances, is usually utilized to assess MLA classification performance. However, accuracy is a poor measure for an imbalanced training dataset. Therefore, two other measures of performance were utilized in this study: area under the receiver operating characteristic (ROC) (AUC) and precision. The ROC curve is a graphical evaluator of a classification algorithm as its discrimination threshold is varied. Assuming two classes: positives and negatives, the ROC curve for an algorithm is created by plotting the recall (aka true positive rate (TPR), sensitivity, or probability of detection) (Equation (3)) against the (1-specificity) (aka false positive rate (FPR) or probability of false alarm) (Equation (4)). The AUC ranges between 0 and 1 where the value of 1 indicates perfect classification performance. Precision (Equation (5)) is an indicator of the number of items correctly identified as positive out of total items identified as positive, thus only examines related observations within a dataset. Furthermore, a confusion matrix, which is a table that reports the number of false positives (FP), false negatives (FN), true positives (TP), and true negatives (TN), allows a more detailed visualization and analysis for the real performance of a classifier. It should be noted here that true and false refer to the correct and incorrect classification, respectively.

(3) $$\mathrm{Recall}=\frac{\mathrm{TP}}{\mathrm{TP}+\mathrm{FN}}$$

(4) $$\mathrm{Specificity}=\frac{\mathrm{TN}}{\mathrm{FP}+\mathrm{TN}}$$

(5) $$\mathrm{Precision}=\frac{\mathrm{TP}}{\mathrm{TP}+\mathrm{FP}}.$$

## 4. Results

### 4.1. Film Mechanical Perfomance

**Figure 1:**
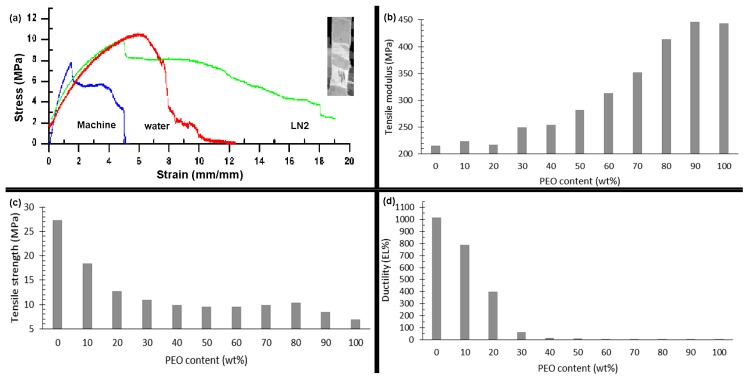
Effect of poly(ethylene oxide) (PEO) content on tensile properties of compression molded poly(ε-caprolactone) (PCL) films: (**a**) stress-strain curves for PCL/PEO 50:50 for different cooling techniques, (**b**) tensile modulus (MPa), (**c**) tensile strength (MPa), and (**d**) ductility (EL%).

**Figure 2:**
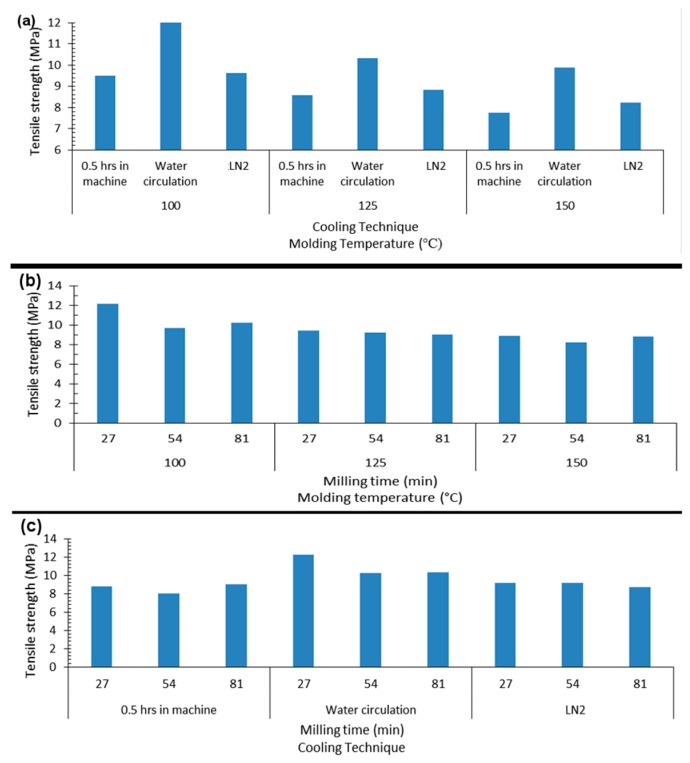
Effect of processing parameters on tensile strength of compression molded PCL/PEO films: (**a**) cooling technique and molding temperature (°C), (**b**) milling time (min) and molding temperature (°C), (**c**) milling time (min) and cooling technique.

**Figure 3:**
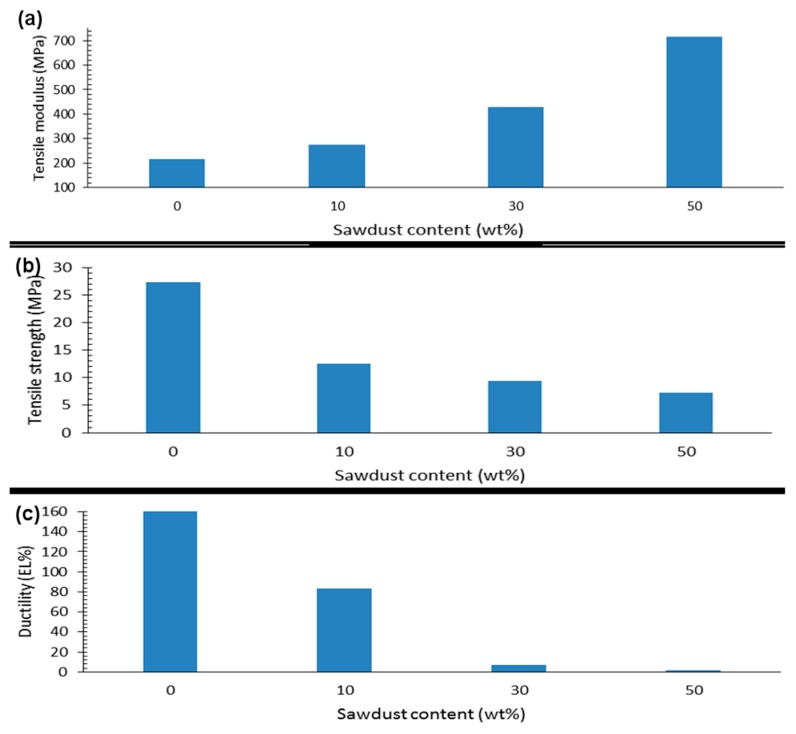
Effect of sawdust content on tensile properties of compression molded PCL-based films: (**a**) tensile modulus (MPa), (**b**) tensile strength (MPa), and (**c**) ductility (EL%).

The mechanical properties of films prepared via extrusion-blow molding and cromilling/compression molding at several processing parameters and material composition were evaluated by tensile tests. Tensile properties were extracted from stress-strain curves. Films showed enormous differences in properties at the different processing parameters. For instance, PCL-based compression-molded films had tensile modulus ranging from 374 to 1270 MPa, tensile strength ranging from 10 to 32 MPa, and ductility from 74 to 1193 elongation (EL)%. Properties were highly nonlinearly and dependent on composition and processing parameters, with complex parameters interactions. Figure 1, Figure 2 and Figure 3 illustrate general property trends, showing the highly nonlinear responses for the cryomilling/compression molding. Comparable figures for the extrusion-blow molding can be found in Figure S2 in the supplementary materials.

Figure 1a displays typical stress-strain curves for a PCL/PEO_50:50_ blend cryomilled to 27 min, molded at 100 °C, and cooled via different means. The curves clearly demonstrate the effect of processing parameters on film mechanical behavior. Figure 2 elaborates more on the effects of each two processing parameters on the tensile strength. The inset in the figure further shows typical sample fracture behavior with increasing stress. Samples demonstrated different levels of plastic extensions, with fracture initiating at different points and propagating along the entire length of the sample till failure. Figure 1b–d summarize the nonlinear effects of PEO content on PCL-based films tensile properties. Similar trends are seen for sawdust effects (Figure 3).

Consequently, the mechanical properties of the polymeric films are highly dependent on the processing parameters, which can therefore be tuned for particular applications. MLA modeling tools are highly capable of solving linear and nonlinear multivariate regression problems. The next section demonstatrates the utilization of MLA for modeling the tensile properties of films produced via the two different processing approaches.

### 4.2. MLA Prediction Perfomance

**Figure 4:**
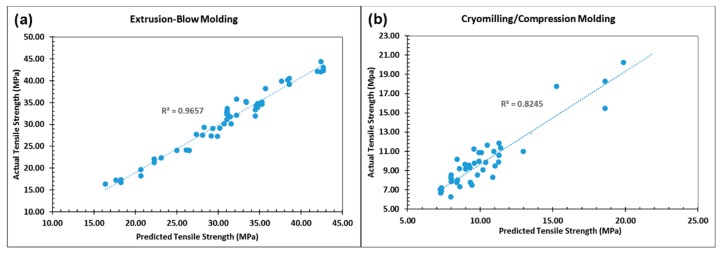
Predicted tensile strength vs. measured tensile stregth by support vector machine (SVM) for: (**a**) extrusion-blow molding, and (**b**) cryomilling/compression molding.

Parameters of the MLA models that presented the best performance with the available datasets are given in Table 1. In order to evaluate and compare the performance of the MLA models an analysis of observed versus predicted strength was conducted in conjunction with statistical metrics R^2^ and MAPE as shown in Table 4. Also, Figure 4a,b demonstrate the comparison graphically based on SVM for the extrusion-blow molding and cryomilling/compression molding, respectively. Consequently, the most suitable algorithm(s) was/were recognized and used to draw conclusions about the studied films fabrication processes. By analyzing these results, the following outcomes can be concluded:

• SVM, kNN, ANN, CART, AB and RF have better performance than the other three algorithms. Different reasons can be behind this good pefrepmance: for ANN, its the powerful framework for modeling nonlinear systems, especially with high dimensional and multivariate datasets [11,18]; related to CART’s, could be its capabilitye of addressing issues of categorical variables in materials research [18]; and for RF, it could be the low variance values [31,42] and ability to handle data sets with higher dimensionality [43].

• SVM exhibited the best performance for both processes studied and based on the both considered criteria. The superiority can be attributed to its excellent modeling of nonlinear relationships without being stuck in local minima [8,12]. This result coincides with related literature since SVM uses the SRM inductive principle that has shown to perform better than the traditional empirical risk minimization inductive principle used, for example, in conventional neural networks [44].

• LR performed poorly in both criterion. This result was foreseeable; as the relationship between the independent variables and the dependent variable for both considered manufacturing processes is highly nonlinear. In addition, LR does not perform well when the number of model parameters is high [39,45].

• A consensus in performance evaluation is noticed between MAPE and R^2^, that is, for a selected MLA, its performance regarding variability and bias has the same trend.

• Generally, the MLA implementation for the extrusion-blow molding resulted in better performance than for the cryomilling/compression molding. This could be attributed to the air entrapment problems associated with compression molding. Lower R^2^ values may indicate that other input parameters should be considered.

### 4.3. MLA Classification Performance

**Figure 5:**
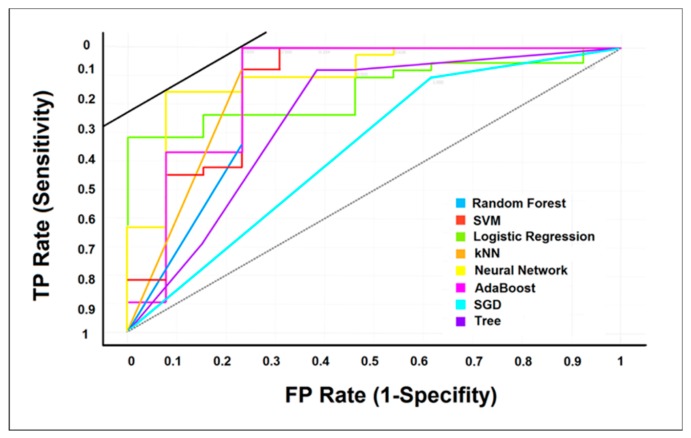
Receiver operating characteristic (ROC) curves for eight machine learning algorithm (MLA).

**Figure 6:**
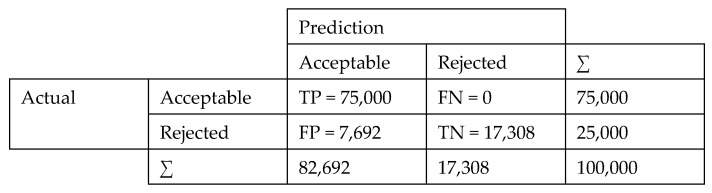
Confusion matrix for k-nearest neighbors (kNN).

Referring to the minimum specification limit for tensile strength (24.13 MPa) set by the ASTM D-882 standard [41], the binary classification ability of MLA to detect nonconforming samples was evaluated for the HDPE films. Table 5 shows the classification performance measures for the tested MLA, including the AUC, accuracy, recall, and precision. The results are also illustrated by the ROC curves (Figure 5). By analyzing these results, the following observations and implications can be concluded:

• The ANN, kNN, AB, SVM, and RF demonstrated very good performance based on the AUC, recall, and precision criteria. For illustration, the 0.929 average AUC for ANN indicates a 92.9% probability that a randomly picked nonconforming film is rated or ranked as more likely to be nonconforming than a randomly picked conforming film [46].

• The high accuracy values are not fully reliable due to the dataset imbalance.

• In order to assess the impact of MLA classification, assume an inspection scenario of HDPE films produced from extrusion-blow with a lot size of 100,000 and 18,000 defectives (using 18% nonconforming percentage as for the studied dataset). Using the kNN confusion matrix shown in Figure 6, 82,692/17,308 films would be identified as conforming/nonconforming; correctly identifying 75,000/17,308 out of the 82,000/18,000 produced films as conforming/nonconforming. Alternatively, if a random sample of 82,692 was selected, only 67,807 (=82,692 × 0.82) films would have been, on average, classified as conforming.

## 5. Conclusions

Recently, soft computing techniques are being utilized by researchers in almost any field. This can be attributed to their unique capabilities in handling complex, nonlinear, categorical, and multi-dimensional prediction and classification problems, where analytical solutions are complicated and time consuming, if not impossible [8,29]. Thus, these tools have gained considerable attention in the materials engineering society. Learning algorithms revealed great advantages of accurately mapping polymers behavior and addressing all types of significant material and processing parameters. The present study has demonstrated that learning algorithms are effective in predicting the tensile strength of polymeric films regardless of the fabrication technique, with the support vector machine (SVM) algorithm demonstrating superior predictive ability. Furthermore, the study has demonstrated the classification capability of these algorithms for sorting films into conforming and nonconforming parts, with several algorithms exhibiting excellent performance.

## Figures and Tables

**Table 1:** Constraints on virgin high-density polyethylene (HDPE) film component proportions and processing parameters.

**HDPE film Component Proportions (wt %)**	virgin HDPE	34 ≤ X_1_ ≤ 70
	recycled HPDE	10 ≤ X_2_ ≤ 40
	CaCO_3_	0 ≤ X_3_ ≤ 20
	copolymer	1 ≤ X_4_ ≤ 6
**Processing Parameters**	CaCO_3_ mean particle size (µm)	Z_1_ = 6, 12
	T1 (°C)	162 ≤ Z_2_ ≤ 196
	T1 (°C)	164 ≤ Z_3_ ≤ 183
	T3 (°C)	163 ≤ Z_4_ ≤ 195
	T4 (°C)	150 ≤ Z_5_ ≤ 188
	mixing speed (rpm)	20 ≤ Z_6_ ≤ 48.2
	bubble drawing speed (m/min)	2.1 ≤ Z_7_ ≤ 6.5

**Table 2:** Compression molding film experimental conditions.

Film Component Proportions			Processing Parameters			
PCL (wt %)	PEO (wt %)	Wood SD (wt %)	Milling Time (min)	Molding Temperature (°C)	Molding Time (min)	Cooling Technique
100–0	0–100	0	27	100	0.5, 5	water
50	50	0	27, 54, 81	100, 125, 150	5	machine, water, LN2
90, 70, 50	0	10, 30, 50	27	100, 125, 150	5	water
45, 35, 25	45, 35, 25	10, 30, 50	27	100, 125, 150	5	water
45, 35, 25	45, 35, 25	10, 30, 50	27	100	0.5	water, LN2

**Table 3:** Selected machine learning algorithm (MLA) parameters.

MLA	MLA Parameters	
	Extrusion-Blow Molding	Cryomilling/Compression Molding
kNN	Number of neighbors: 11 Metric: Mahalanobis Weight: distance	Number of neighbors: 21 Metric: Mahalanobis Weight: distance
DT (CART)	Pruning: at least three instances in internal nodes, maximum depth 100 Splitting: stop splitting when majority reaches 95% (classification only) Binary trees: yes	Pruning: at least three instances in leaves (terminal nodes), at least three instances in internal nodes, maximum depth 100 Splitting: stop splitting when majority reaches 95% (classification only) Binary trees: no
RF	Number of trees: 14 Maximal number of considered features: unlimited Fixed random seed: three (four for classification) Maximal tree depth: unlimited Stop splitting nodes with maximum instances: (two for classification)	Number of trees: 21 Maximal number of considered features: unlimited Fixed random seed: three Maximal tree depth: six Stop splitting nodes with maximum instances: 5
AB	Base estimator: tree Number of estimators: 45 (100 for classification) Algorithm (classification): Samme.r Loss (regression): linear	Base estimator: tree Number of estimators: 4 Algorithm (classification): Samme.r Loss (regression): linear
SVM	SVM type: SVM, C (penalty parameter) = 100.8, ε (kernel coefficient) = 1.5 Kernel: RBF, exp.(−2.12|x−y|^2^) Numerical tolerance: 0.001 Iteration limit: 100	SVM type: SVM, C = 16.30, ε = 1.1 Kernel: RBF, exp.(−0.35|x−y|^2^) Numerical tolerance: 0.001 Iteration limit: 100
SGD	Classification loss function: hinge Regression loss function: squared loss Regularization: none (“elastic net” for classification) Regularization strength (α): 0.00053 (for classification) Elastic net mixing parameter (L1 ratio): 0.16100 (for classification) Learning rate: Inverse scaling (“optimal” for classification) Initial learning rate (η_0_): 0.0001 Inverse scaling exponent (t): 0.0104 Shuffle data after each iteration: yes	Classification loss function: Huber Epsilon (ε) for classification: 0.92 Regression loss function: squared loss Regularization: elastic net Regularization strength (α): 0.05 Elastic Net mixing parameter (L1 ratio): 0.1 Learning rate: inverse scaling Initial learning rate (η0): 0.0008 Inverse scaling exponent (t): 0.0142 Shuffle data after each iteration: yes
ANN	Hidden layers: 80, 80 Activation: tanh (“ReLu” for classification) Solver: L-BFGS-B (“Adam” for classification) Alpha: 0.0001 Max iterations: 300	Hidden layers: 50, 50 Activation: logistic Solver: L-BFGS-B Alpha: 0.0001 Max iterations: 300
LR	Regularization: no regularization (only for regression)	Regularization: no regularization
LoR	Regularization: lasso (L1), C = 0.8 (Only for classification)	-

**Table 4:** MLA prediction evaluation for the film production processes.

MLA	R^2^ (%)		MAPE (%)	
	Extrusion-Blow Molding	Cryomilling/Compression Molding	Extrusion-Blow Molding	Cryomilling/Compression Molding
RF	87	76	7	11
SVM	96	81	4	11
LR	24	76	19	11
kNN	94	73	4	13
ANN	93	73	4	13
ABt	91	71	5	14
SGD	24	77	19	11
CART	94	73	4	13

**Table 5:** MLA classification evaluation for the extrusion-blow molding process.

MLA	AUC	Accuracy ^1^	Precision ^2^	Recall
ANN	0.901	0.808	0.796	1
kNN	0.876	0.923	0.907	1
AdaBoost	0.872	0.942	0.929	1
SVM	0.862	0.923	0.907	1
LoR	0.852	0.750	0.771	0.949
RF	0.840	0.923	0.907	1
CART	0.754	0.827	0.857	0.923
SGD	0.641	0.769	0.814	0.897

^1^: Accuracy is the proportion of correctly classified instances, given by: $\mathrm{Accuracy}=\frac{\mathrm{TP}+\mathrm{TN}}{\mathrm{TP}+\mathrm{FP}+\mathrm{TN}+\mathrm{FN}}$; ^2^: Precision is the proportion of TP among instances classified as positive.

## Supplementary Materials

The following are available online at https://www.mdpi.com/1996-1944/12/9/1475/s1, Figure S1: Screw of the extruder, Figure S2: Relationships of input parameters vs. tensile strength for the extrusion process, Table S1: Extrusion HDPE film experimental data set, Table S2: Compression molding film experimental data set.
