# Supplementary material for: Machine Learning Models for Predicting and Classifying the Tensile Strength of Polymeric Films Fabricated via Different Production Processes
Source: Materials (Basel). 2019 May 7;12(9):1475. doi: 10.3390/ma12091475 (PMC6539900; doi:10.3390/ma12091475)
Supplement: Supplementary file 1 [file materials-12-01475-s001.pdf]

materials

Supplementary Materials

Machine Learning Models for Predicting and Classifying the Tensile Strength of Polymeric Films Fabricated via Different Production Processes

Safwan Altarazi [1,*], Rula Allaf [2] and Firas Alhindawi [3]

Industrial Engineering Department, German Jordanian University, 11180 Amman, Jordan;
rula.alalf@gju.edu.jo (R.A.); firas.alhindawi@gju.edu.jo (F.A.)
* Correspondence: safwan.altarazi@gju.edu.jo; Tel.: +96264294522

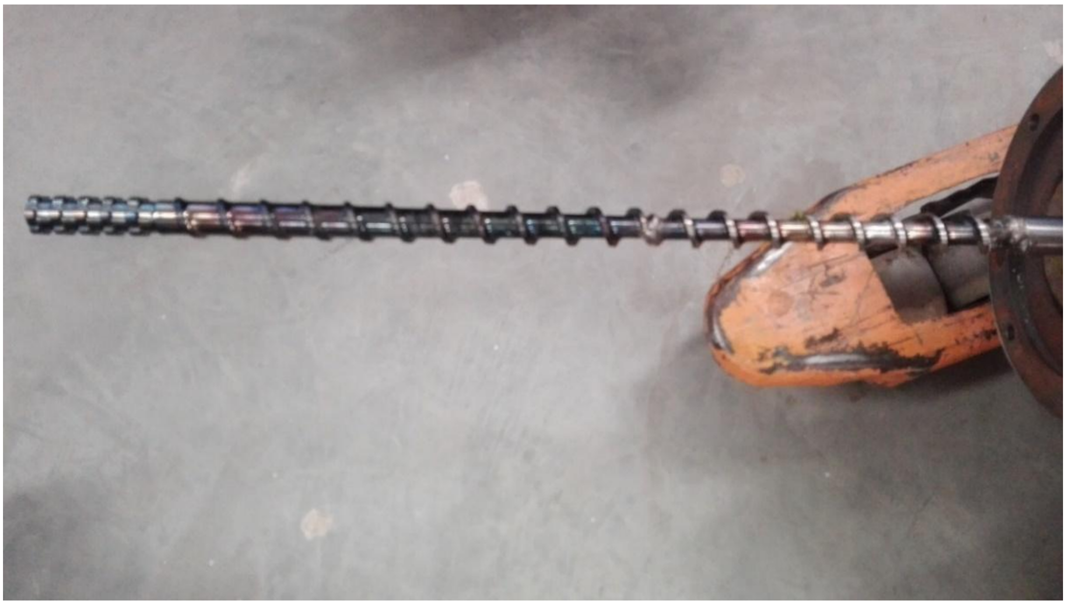

Figure S1. The screw of the extruder

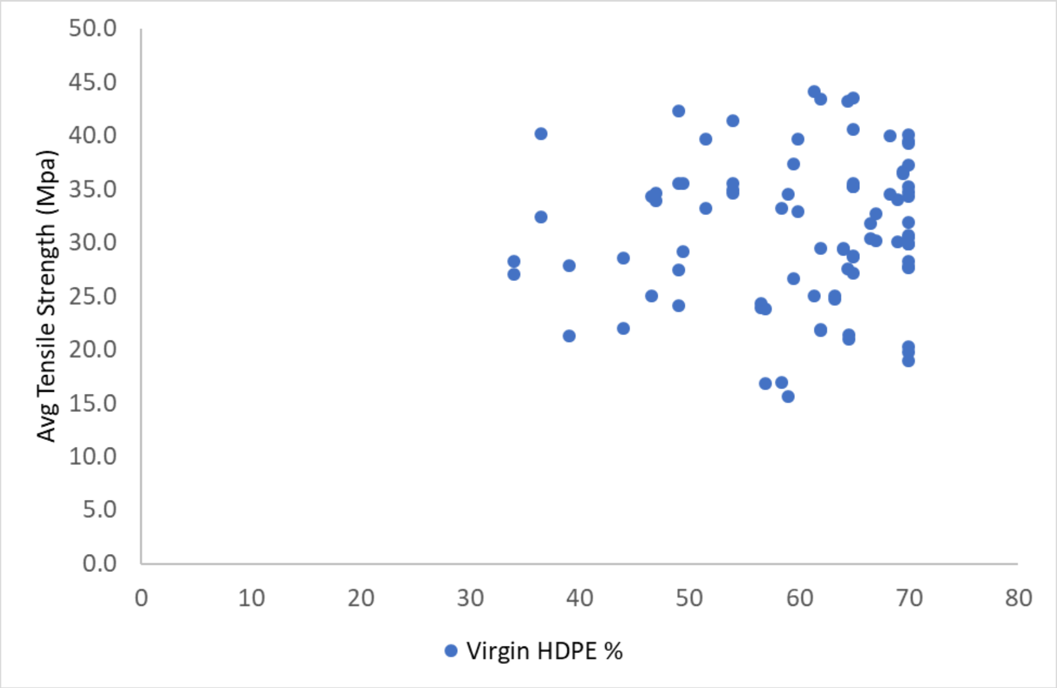
50.0
45.0
40.0
35.0
30.0
25.0
20.0
15.0
10.0
5.0
0.0
Avg Tensile Strength (Mpa)
0
10
20
30
40
50
60
70
80
Virgin HDPE %

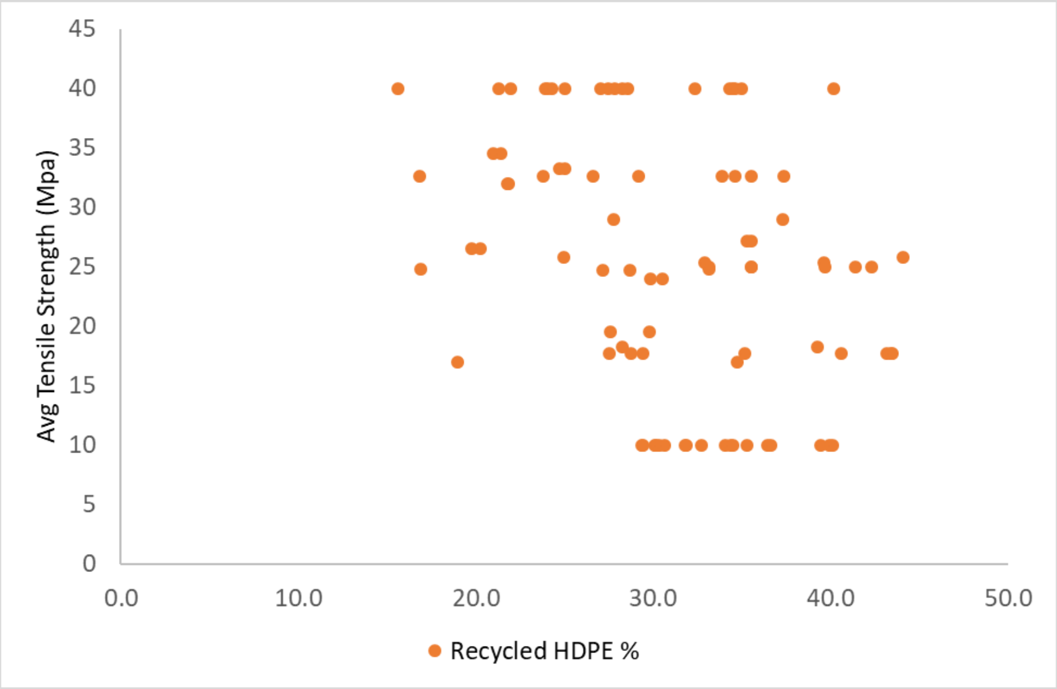
45
40
35
30
25
20
15
10
5
0
Avg Tensile Strength (Mpa)
0.0
10.0
20.0
30.0
40.0
50.0
Recycled HDPE %

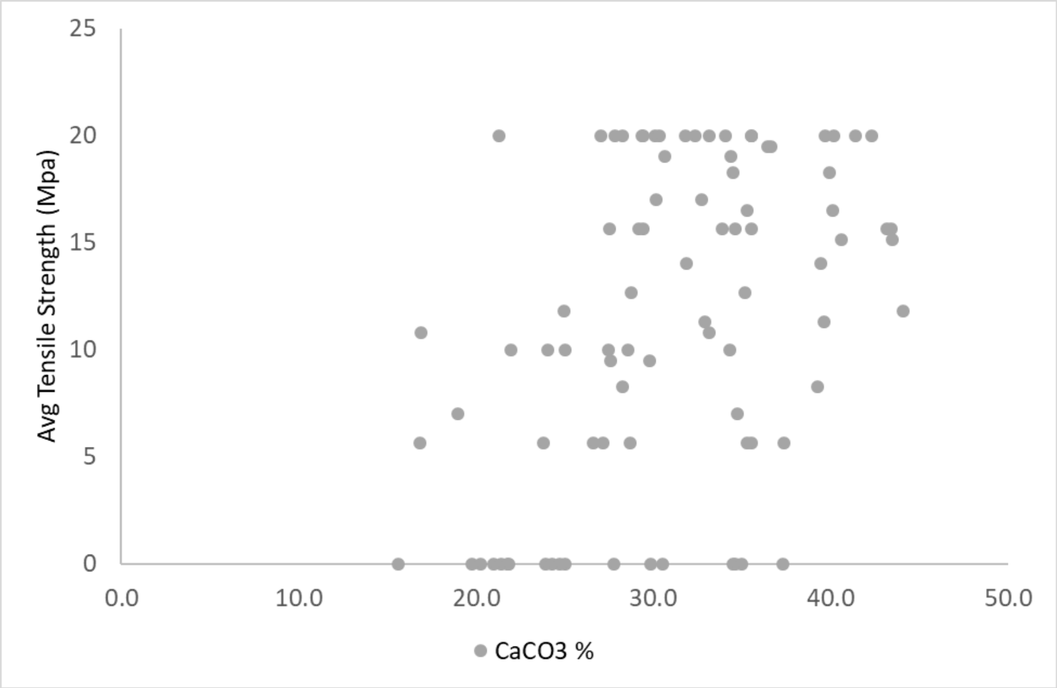
Avg Tensile Strength (Mpa)
25
20
15
10
5
0
0.0
10.0
20.0
30.0
40.0
50.0
CaCO3 %

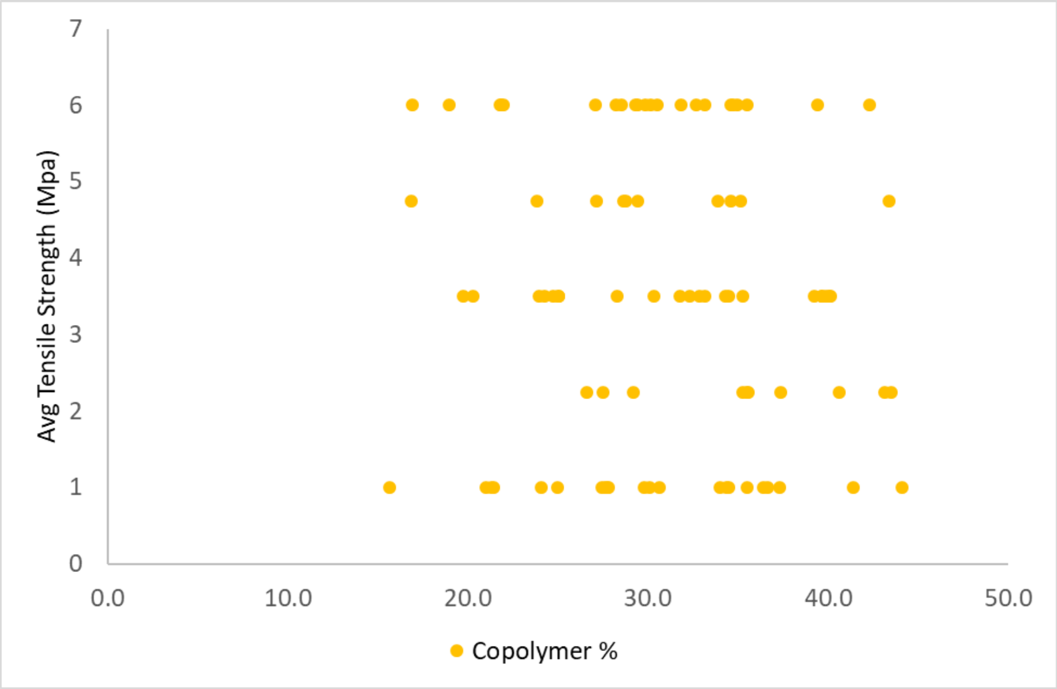
Avg Tensile Strength (Mpa)
7
6
5
4
3
2
1
0
0.0
10.0
20.0
30.0
40.0
50.0
Copolymer %

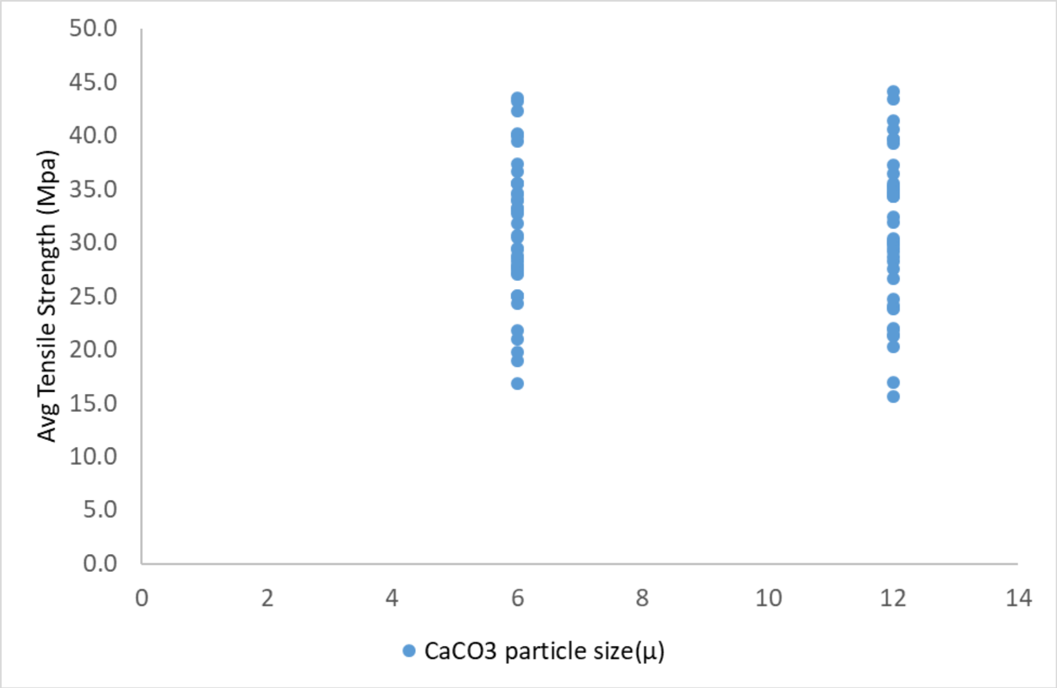
Avg Tensile Strength (Mpa)
50.0
45.0
40.0
35.0
30.0
25.0
20.0
15.0
10.0
5.0
0.0
0
2
4
6
8
10
12
14
CaCO3 particle size(µ)

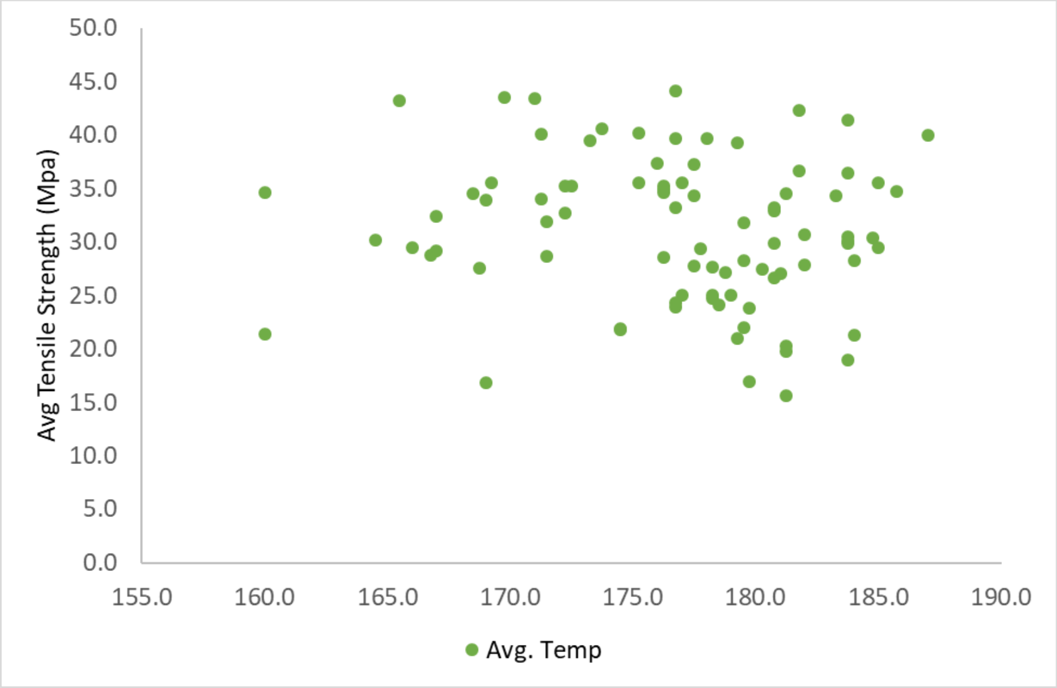
Avg Tensile Strength (Mpa)
50.0
45.0
40.0
35.0
30.0
25.0
20.0
15.0
10.0
5.0
0.0
155.0
160.0
165.0
170.0
175.0
180.0
185.0
190.0
Avg. Temp

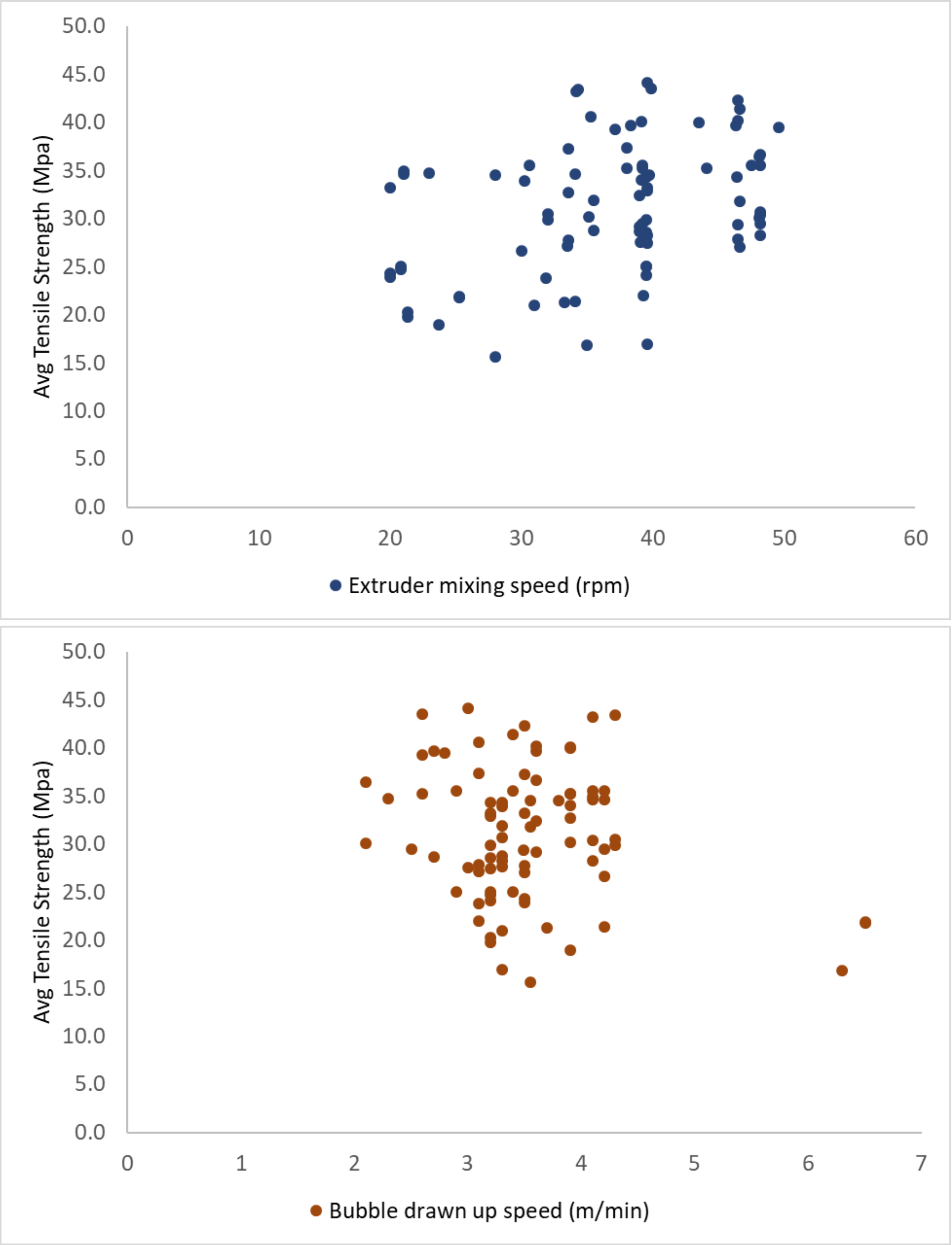

Figure S2. Relationships of input parameters vs. tensile strength for the extrusion process.

materials

MDPI

Table S1. Extrusion HDPE film experimental data set.

Sample number	HDPE film component proportions (wt%)					Processing parameters						Output
	Virgin HDPE (%)	Recycled HDPE (%)	$CaCO_3$ (%)	Copolymer (%)	$CaCO_3$ particle size (μ)	Extruder heaters temperature (°C)				Extruder mixing speed (rpm)	Bubble drawn up speed (m/min)	Tensile Strength (MPa)
						1st	2nd	3rd	4th			
1	68.25	10	18.25	3.5	12	177	164	176	157	39.7	3.8	34.50 ± 2.34
2	56.95	32.65	5.65	4.75	6	166	170	179	161	35	6.3	16.85 ± 1.82
3	46.95	32.65	15.65	4.75	6	181	164	170	161	30.2	3.3	33.90 ± 5.13
4	61.4	25.8	11.8	1	6	187	174	186	161	39.5	2.9	24.98 ± 3.27
5	59	40	0	1	12	170	180	190	185	28	3.55	15.63 ± 2.11
6	70	17	7	6	6	186	180	188	181	23.67	3.9	18.95 ± 2.13
7	70	24	0	6	12	176	180	191	188	32	4.3	29.85 ± 1.57
8	49.45	32.65	15.65	2.25	6	182	164	170	161	30.6	4.1	35.55 ± 5.85
9	46.5	40	10	3.5	12	178	174	187	171	39.53	3.2	34.30 ± 6.41
10	67	10	17	6	6	180	164	180	165	33.56	3.9	32.70 ± 2.62
11	70	26.5	0	3.5	6	178	175	189	183	21.3	3.2	19.75 ± 1.82
12	59.9	25.3	11.3	3.5	6	188	174	187	174	39.6	3.2	32.88 ± 5.52
13	64.5	34.5	0	1	6	173	174	188	182	31	3.3	21.02 ± 2.93
14	64.5	34.5	0	1	12	162	164	163	151	34.11	4.2	21.42 ± 4.46
15	46.95	32.65	15.65	4.75	12	162	164	163	151	34.1	4.2	34.58 ± 4.83
16	70	19.5	9.5	1	6	187	174	181	171	39.5	3.3	27.60 ± 3.46
17	67	10	17	6	12	176	165	167	150	35.14	3.9	30.13 ± 2.41
18	69.5	10	19.5	1	12	187	180	187	181	48.11	2.1	36.43 ± 2.27
19	69	10	20	1	12	187	180	187	181	48.11	2.1	30.07 ± 1.05
20	64.95	27.15	5.65	2.25	6	179	165	179	178	39.2	2.9	35.52 ± 1.60
21	44	40	10	6	6	176	174	183	172	39.5	3.2	28.55 ± 4.14
22	70	29	0	1	12	172	175	184	179	33.6	3.5	37.28 ± 2.57
23	64.95	24.65	5.65	4.75	12	180	165	174	167	39	2.7	28.67 ± 3.37
24	66.5	10	20	3.5	12	192	180	185	182	48.2	4.1	30.33 ± 2.13

25	61.95	17.65	15.65	4.75	12	181	164	179	160	34.3	4.3	43.40 ± 3.69
26	68.25	10	18.25	3.5	6	190	183	195	180	43.52	3.9	39.92 ± 2.08
27	70	10	14	6	12	186	170	166	164	35.5	3.3	31.83 ± 6.09
28	39	40	20	1	12	192	180	184	180	33.3	3.7	21.32 ± 1.90
29	64.95	17.65	15.15	2.25	12	184	173	170	168	35.3	3.1	40.60 ± 5.56
30	64	10	20	6	12	196	180	185	179	48.2	4.2	29.43 ± 2.79
31	54	25	20	1	6	194	180	185	181	48.18	4.2	35.50 ± 2.26
32	34	40	20	6	12	192	180	185	179	48.2	4.1	28.25 ± 2.41
33	64.45	17.65	15.65	2.25	6	177	164	164	157	34.13	4.1	43.17 ± 2.88
34	56.5	40	0	3.5	12	167	175	185	180	20	3.5	23.93 ± 1.21
35	70	10	16.5	3.5	6	180	164	178	163	39.11	3.9	40.08 ± 5.18
36	59	40	0	1	6	170	180	190	185	28	3.55	34.47 ± 3.06
37	70	26.5	0	3.5	12	178	175	189	183	21.3	3.2	20.28 ± 2.44
38	69	10	20	1	6	180	164	178	163	39.11	3.9	34.03 ± 1.54
39	58.4	24.8	10.8	6	6	190	174	186	173	39.55	3.2	33.15 ± 1.43
40	64.95	17.65	12.65	4.75	12	186	171	184	164	44.11	3.9	35.17 ± 1.95
41	63.25	33.25	0	3.5	6	172	175	186	180	20.8	3.2	25.03 ± 3.15
42	70	29	0	1	6	172	175	184	179	33.6	3.5	27.77 ± 1.51
43	46.5	40	10	3.5	6	183	174	186	173	39.5	3.4	25.03 ± 4.85
44	51.5	25	20	3.5	6	167	175	185	180	20	3.5	33.17 ± 4.95
45	70	19.5	9.5	1	12	184	174	188	177	39.5	3.2	29.82 ± 1.74
46	49	40	10	1	12	184	174	185	171	39.5	3.2	24.07 ± 1.54
47	56.5	40	0	3.5	6	167	175	185	180	20	3.5	24.27 ± 2.25
48	70	18.25	8.25	3.5	12	185	174	187	171	37.11	2.6	39.23 ± 1.95
49	64.95	27.15	5.65	2.25	12	176	165	178	171	38	2.6	35.27 ± 2.00
50	63.25	33.25	0	3.5	12	172	175	186	180	20.8	3.2	24.73 ± 1.93
51	70	10	19	1	12	187	180	184	182	46.4	3.3	34.35 ± 1.43
52	54	40	0	6	6	166	175	184	180	21	4.1	34.58 ± 4.19
53	70	18.25	8.25	3.5	6	186	174	186	172	39.6	3.3	28.28 ± 1.88
54	54	25	20	1	12	190	180	185	180	46.6	3.4	41.42 ± 6.51
55	64.95	17.65	15.15	2.25	6	180	173	166	160	39.9	2.6	43.48 ± 2.73
56	66.5	10	20	3.5	6	185	180	185	168	46.6	3.55	31.78 ± 6.18

57	59.45	32.65	5.65	2.25	6	181	170	181	172	38	3.1	37.37 ± 2.29
58	64	10	20	6	6	182	180	184	165	46.5	3.49	29.33 ± 2.33
59	61.95	17.65	15.65	4.75	6	176	164	168	156	39.17	2.5	29.43 ± 1.95
60	44	40	10	6	12	185	174	185	174	39.3	3.1	21.98 ± 0.98
61	64.45	17.65	15.65	2.25	12	180	164	177	154	39.09	3	27.50 ± 3.93
62	51.5	25	20	3.5	12	181	180	184	162	46.3	3.6	39.68 ± 2.05
63	54	40	0	6	12	166	175	184	180	21	4.1	34.95 ± 3.73
64	70	10	16.5	3.5	12	186	164	177	162	39.2	3.9	35.27 ± 2.91
65	49	40	10	1	6	188	174	186	173	39.6	3.2	27.45 ± 2.33
66	61.4	25.8	11.8	1	12	186	174	185	162	39.59	3	44.10 ± 2.46
67	49	25	20	6	12	178	180	186	164	47.5	3.4	35.50 ± 3.46
68	59.9	25.3	11.3	3.5	12	196	174	184	158	38.3	2.7	39.65 ± 4.14
69	64.95	17.65	12.65	4.75	6	177	170	165	155	35.49	3.3	28.75 ± 3.35
70	58.4	24.8	10.8	6	12	191	174	184	170	39.6	3.3	16.92 ± 1.02
71	36.5	40	20	3.5	6	177	180	182	162	46.5	3.6	40.15 ± 0.69
72	39	40	20	1	6	180	180	190	178	46.5	3.1	27.83 ± 3.92
73	70	24	0	6	6	176	180	191	188	32	4.3	30.52 ± 4.56
74	49	25	20	6	6	182	180	185	180	46.5	3.5	42.30 ± 5.72
75	70	10	19	1	6	192	180	184	172	48.16	3.3	30.63 ± 3.63
76	70	10	14	6	6	185	170	177	161	49.6	2.8	39.43 ± 2.96
77	59.45	32.65	5.65	2.25	12	187	176	179	181	30	4.2	26.60 ± 2.89
78	56.95	32.65	5.65	4.75	12	185	176	182	176	31.9	3.1	23.82 ± 3.95
79	70	17	7	6	12	194	180	191	178	23	2.3	34.72 ± 3.98
80	62	32	0	6	12	171	175	186	166	25.3	6.5	21.85 ± 2.78
81	34	40	20	6	6	180	180	184	180	46.6	3.5	27.07 ± 2.00
82	49.45	32.65	15.65	2.25	12	176	164	172	156	39	3.6	29.17 ± 2.29
83	36.5	40	20	3.5	12	176	164	172	156	39	3.6	32.35 ± 1.88
84	62	32	0	6	6	171	175	186	166	25.3	6.5	21.78 ± 2.19
85	64.95	24.65	5.65	4.75	6	182	176	181	176	33.5	3.1	27.15 ± 3.00
86	69.5	10	19.5	1	6	193	180	185	169	48.2	3.6	36.65 ± 1.57

Table S2. Compression molding film experimental data set

**Film component proportions (wt%)**			**Processing parameters**				**Output**			
**PCL**	**PEO**	**Wood SD**	**Milling time (min)**	**Molding Temperature (°C)**	**Molding Time (min)**	**Cooling Technique**	**Thickness (mm)**	**Tensile modulus (MPa)**	**Tensile strength (MPa)**	**Ductility (%EL)**
100	0	0	27	100	0.5	water	0.16±0.01	208±46	27.90±3.99	1135.96±95.17
90	10	0	27	100	0.5	water	0.16±0.01	209±26	19.33±4.04	860.83±173.32
80	20	0	27	100	0.5	water	0.16±0.01	195±23	13.16±1.31	654±78.1
70	30	0	27	100	0.5	water	0.15±0.02	235±14	10.79±1.77	55.81±23.66
60	40	0	27	100	0.5	water	0.12±0.01	200±8	8.57±3.36	20.23±1.10
50	50	0	27	100	0.5	water	0.16±0.01	295±37	10.78±0.70	18.51±5.64
40	60	0	27	100	0.5	water	0.16±0.01	342±9	9.31±1.22	8.23±1.66
30	70	0	27	100	0.5	water	0.15±0.00	357±44	9.39±1.67	8.70±4.09
20	80	0	27	100	0.5	water	0.15±0.00	377±29	8.96±0.99	5.25±0.69
10	90	0	27	100	0.5	water	0.15±0.02	415±33	7.22±1.02	2.64±0.66
0	100	0	27	100	0.5	water	0.16±0.01	460±82	7.19±1.03	1.67±0.38
100	0	0	27	100	5	water	0.10±0.00	233±31	27.25±1.28	895.00±24.58
90	10	0	27	100	5	water	0.10±0.01	238±23	17.49±1.76	717.67±78.81
80	20	0	27	100	5	water	0.11±0.01	240±32	12.35±1.42	141.00±184.58
70	30	0	27	100	5	water	0.10±0.00	264±13	11.10±1.31	66.77±27.38
60	40	0	27	100	5	water	0.11±0.01	309±30	11.22±0.64	13.04±1.66
40	60	0	27	100	5	water	0.11±0.01	284±63	9.87±2.19	5.84±0.98
30	70	0	27	100	5	water	0.11±0.00	347±75	10.42±1.90	6.22±1.03
20	80	0	27	100	5	water	0.12±0.01	450±105	11.74±1.72	4.84±0.58
10	90	0	27	100	5	water	0.11±0.00	478±98	9.68±2.06	3.28±1.62
0	100	0	27	100	5	water	0.11±0.00	419±34	6.51±0.23	2.00±0.57
50	50	0	27	100	5	Machine	0.11±0.01	315±19	10.03±1.95	9.11±4.54
50	50	0	27	100	5	Water	0.12±0.00	326±55	11.20±1.23	8.88±1.97
50	50	0	27	100	5	LN2	0.13±0.01	319±13	10.42±0.45	19.3±7.05
50	50	0	27	125	5	Machine	0.09±0.01	247±23	7.96±1.41	10.64±8.15
50	50	0	27	125	5	Water	0.11±0.00	276±20	11.11±0.37	11.49±1.91
50	50	0	27	125	5	LN2	0.12±0.01	298±6.51	9.31±1.04	14.29±4.85
50	50	0	27	150	5	Machine	0.08±0.00	218±23	8.29±0.38	6.79±1.59

50	50	0	27	150	5	Water	0.09±0.01	250±24	10.63±1.61	9.88±3.30
50	50	0	27	150	5	LN2	0.10±0.01	251±39	7.79±0.77	4.79±1.55
50	50	0	54	100	5	Machine	0.11±0.01	292±38	8.37±0.80	7.80±1.04
50	50	0	54	100	5	Water	0.12±0.00	339±36	11.46±0.17	9.86±1.82
50	50	0	54	100	5	LN2	0.13±0.01	271±25	9.32±0.48	5.65±1.61
50	50	0	54	125	5	Machine	0.09±0.01	278±40	8.62±0.90	5.57±2.01
50	50	0	54	125	5	Water	0.11±0.01	259±4	10.10±0.47	6.95±0.64
50	50	0	54	125	5	LN2	0.11±0.01	253±14	9.04±0.61	12.17±2.98
50	50	0	54	150	5	Machine	0.08±0.01	251±49	7.18±0.85	5.83±3.43
50	50	0	54	150	5	Water	0.10±0.00	242±25	9.29±0.33	6.15±1.65
50	50	0	81	100	5	Machine	0.10±0.01	244±104	10.06±1.31	7.90±2.70
50	50	0	81	100	5	Water	0.12±0.01	286±23	11.48±0.23	8.55±3.95
50	50	0	81	100	5	LN2	0.13±0.01	350±15	9.13±1.51	9.26±4.63
50	50	0	81	125	5	Machine	0.10±0.00	278±18	9.12±0.82	13.67±6.00
50	50	0	81	125	5	Water	0.12±0.00	248±32	9.75±0.60	10.29±3.56
50	50	0	81	125	5	LN2	0.12±0.01	360±44	8.16±1.36	8.56±6.08
50	50	0	81	150	5	Machine	0.08±0.00	251±15	7.82±0.97	9.57±4.72
50	50	0	81	150	5	Water	0.10±0.01	295±47	9.76±0.89	6.06±1.24
50	50	0	81	150	5	LN2	0.09±0.00	287±54	8.91±0.97	26.75±16.62
90	0	10	27	100	5	water	0.11±0.03	318±80	14.57±2.95	60.22±48.29
90	0	10	27	125	5	water	0.11±0.02	241±65	11.95±1.00	167.65±249.03
90	0	10	27	150	5	water	0.08±0.01	269±17	11.12±1.33	22.74±11.62
70	0	30	27	100	5	water	0.13±0.01	493±79	10.28±1.53	6.36±2.36
70	0	30	27	125	5	water	0.12±0.00	460±35	9.60±0.56	7.37±4.49
70	0	30	27	150	5	water	0.10±0.01	337±168	8.20±1.72	8.27±4.15
50	0	50	27	100	5	water	0.19±0.01	707±55	8.13±2.26	2.57±1.85
50	0	50	27	125	5	water	0.18±0.02	734±243	6.89±0.15	1.68±0.68
50	0	50	27	150	5	water	0.15±0.01	707±133	6.79±0.46	1.45±0.81
45	45	10	27	100	5	water	0.13±0.02	333±55	8.73±0.60	4.38±0.92
45	45	10	27	125	5	water	0.12±0.01	290±56	8.92±0.63	4.76±0.94
45	45	10	27	150	5	water	0.11±0.03	292±18	8.27±1.07	4.40±1.12
35	35	30	27	100	5	water	0.14±0.02	576±107	8.77±0.64	2.52±0.42

35	35	30	27	125	5	water	0.127±0.01	474±110	7.34±1.42	2.37±0.55
35	35	30	27	150	5	water	0.12±0.01	510±12	8.36±0.56	2.43±0.35
25	25	50	27	100	5	water	0.18±0.01	806±125	6.77±0.13	1.39±0.01
25	25	50	27	125	5	water	0.15±0.016	825±169	5.19±2.67	1.02±0.53
25	25	50	27	150	5	water	0.15±0.01	802±228	6.25±1.67	1.18±0.56
45	45	10	27	100	0.5	water	0.17±0.00	413±12	9.15±1.66	4.43±1.39
45	45	10	27	100	0.5	LN2	0.19±0.01	501±11	8.75±0.83	3.47±1.09
35	35	30	27	100	0.5	water	0.15±0.01	784±84	9.00±1.71	2.10±0.14
35	35	30	27	100	0.5	LN2	0.20±0.01	658±32	8.17±0.94	3.15±0.50
25	25	50	27	100	0.5	water	0.20±0.03	1232±54	6.44±2.19	1.30±0.28
25	25	50	27	100	0.5	LN2	0.23±0.02	1030±2	7.93±0.05	2.50±0.57
